# High-Fat Diet Reduces the Formation of Butyrate, but Increases Succinate, Inflammation, Liver Fat and Cholesterol in Rats, while Dietary Fibre Counteracts These Effects

**DOI:** 10.1371/journal.pone.0080476

**Published:** 2013-11-13

**Authors:** Greta Jakobsdottir, Jie Xu, Göran Molin, Siv Ahrné, Margareta Nyman

**Affiliations:** Department of Applied Nutrition and Food Chemistry, Lund University, Lund, Sweden; Virginia Tech, United States of America

## Abstract

**Introduction:**

Obesity is linked to type 2 diabetes and risk factors associated to the metabolic syndrome. Consumption of dietary fibres has been shown to have positive metabolic health effects, such as by increasing satiety, lowering blood glucose and cholesterol levels. These effects may be associated with short-chain fatty acids (SCFAs), particularly propionic and butyric acids, formed by microbial degradation of dietary fibres in colon, and by their capacity to reduce low-grade inflammation.

**Objective:**

To investigate whether dietary fibres, giving rise to different SCFAs, would affect metabolic risk markers in low-fat and high-fat diets using a model with conventional rats for 2, 4 and 6 weeks.

**Material and Methods:**

Conventional rats were administered low-fat or high-fat diets, for 2, 4 or 6 weeks, supplemented with fermentable dietary fibres, giving rise to different SCFA patterns (pectin – acetic acid; guar gum – propionic acid; or a mixture – butyric acid). At the end of each experimental period, liver fat, cholesterol and triglycerides, serum and caecal SCFAs, plasma cholesterol, and inflammatory cytokines were analysed. The caecal microbiota was analysed after 6 weeks.

**Results and Discussion:**

Fermentable dietary fibre decreased weight gain, liver fat, cholesterol and triglyceride content, and changed the formation of SCFAs. The high-fat diet primarily reduced formation of SCFAs but, after a longer experimental period, the formation of propionic and acetic acids recovered. The concentration of succinic acid in the rats increased in high-fat diets with time, indicating harmful effect of high-fat consumption. The dietary fibre partly counteracted these harmful effects and reduced inflammation. Furthermore, the number of *Bacteroides* was higher with guar gum, while noticeably that of *Akkermansia* was highest with the fibre-free diet.

## Introduction

Obesity/overweight is a serious health risk and related to many metabolic diseases, such as type 2 diabetes, insulin resistance and coronary heart disease, stroke and cancer [1], [2]. Another obesity-associated disease, increasing in prevalence, is non-alcoholic fatty liver disease (NAFLD) and its progressive form, non-alcoholic steatohepatitis. As the optimal treatment still remains unknown [3], finding ways to prevent NAFLD and to reduce the prevalence are of great importance. The management of the metabolic syndrome and other obesity-associated diseases has been shown to be facilitated by an increased consumption of dietary fibre [4]–[7]. However, as the dietary fibre intake is lower than recommended in western countries and also because dietary fibre comprises a complex group of substances [8], [9], it is of great interest to find dietary fibres with the most favourable effects, and also to understand the mechanisms behind these effects.

It is generally accepted that soluble fibres improve glycaemia and insulin sensitivity in both healthy and diabetic subjects, and oat β-glucan may lower plasma cholesterol levels [4], [10] by entrapping bile acids or reducing motility in the upper part of the intestinal tract [11]. Dietary fibre has also been shown to reduce gastric emptying rate and insulin secretion and, thereby increase satiety and reduce energy intake. An alternative and increasingly proposed mechanism, not studied to any great extent, may be alterations in the short-chain fatty acids (SCFAs) formed in colon and changes in gut microbiota composition [12]. SCFAs *per se* have been shown to have many positive health effects, such as reducing pro-inflammatory state, increasing insulin sensitivity and improving satiety [7], [13].

Different fibres give rise to different amounts and patterns of the main SCFAs: acetic, propionic and butyric acids. Pectin forms high amounts of acetic acid, while guar gum yields propionic acid and β-glucan, fructo-oligosaccharides, some types of resistant starch and mixtures of dietary fibres form high amounts of butyric acid [14], [15]. Other factors that can affect the formation of SCFAs are the composition of the colonic microbiota, the type of glycosidic linkages, and the transit time through the gut. The various types of SCFAs have been connected with different physiological effects. Butyric acid is an important energy source for the colonic epithelial cells and has been shown to inhibit growth of cancer cells *in vitro* [16]. Patients with inflammatory bowel disease have a reduced butyrate metabolism and uptake compared with healthy controls [17]. Furthermore, some SCFAs, especially propionic acid, have been shown to lower cholesterol levels [18] and to have a beneficial effect on glucose and lipid metabolism [19], [20]. Butyric acid is also thought to be involved in lipid metabolism, by regulating and slowing down fat transport from the intestine [21], [22]. On the other hand, acetic acid is one of the primary substrates for cholesterol synthesis in the liver via acetyl-CoA, therefore a decreased acetic:propionic acid ratio is preferred. Since the pattern of SCFAs formed is dependent on several food factors, the cholesterol metabolism, for example, could possibly be controlled by diet [23]. Altered cholesterol level is one of the key components of the metabolic syndrome [24]. Other components of the metabolic syndrome are obesity, high blood pressure and high fasting blood glucose levels [24]. Dietary fibres may also modulate the colonic microbiota to a more protective one, and alter systemic and mucosal immune response [25]. Obesity changes the composition of the microbiota, which has been suggested to be connected with body weight [26], and conventionalization of normal caecal microbiota to germ-free C57BL/6 mice resulted in 60% more body fat [27]. Obese mice have more *Fermicutes* and fewer *Bacteroidetes*, when compared with their wild-type littermates [26]. Exposure time is also important; adaption of the microbiota to a changed diet may take time, and the proportion of butyric acid has been shown to increase with time [28].

Obesity and high-fat diet is thought to trigger low-grade inflammation and possibly the development of obesity-associated diseases [29], [30]. Propionic and butyric acids have been shown to suppress pro-inflammatory cytokines and might therefore be important [31].

In the present study, two fermentable dietary fibres, pectin (acetic acid producer) and guar gum (propionic acid producer), and a mixture of the two (butyric acid producer), were chosen [32]-[34]. Pectin is a polysaccharide made up of a main chain of mainly galacturonic acid units, and galactose and arabinose as side chains [32], [35]. Guar gum is a galactomannan of high viscosity [33]. The dietary fibre types included in this study are only a small part of a normal diet and are only used marginally in the food industry, but were chosen because of their ability to give rise to different SCFA profiles. The aim was to clarify whether dietary fibres, known to give rise to different SCFAs, would affect metabolic risk markers in low-fat and high-fat diets using a model with conventional rats. The high-fat content was administered to trigger low-grade inflammation, and the effect of time was investigated (2, 4 and 6 weeks). Formation of carboxylic acids (CA; SCFA along with lactic and succinic acid) in the hindgut of rats and SCFAs in serum, metabolic risk markers (amount of fat, cholesterol and triglycerides in the liver, plasma cholesterol and some cytokines in serum) and the caecal microbiota were analysed. The weight gain was documented.

## Materials and Methods

### Materials

Fermentable dietary fibres, pectin (esterification 70–75%) isolated from apples, and guar gum (viscosity 3.025 mPaS at 1% (w/v) and 25°C), isolated from guar bean (Sigma Aldrich, St. Louis, MO, USA), individually or as a mixture, were included in low-fat diet (LFD) or high-fat diet (HFD) (Table 1).

**Table 1:** Diet composition (g/kg, dwb) and percentage energy (E%) of the diets.

	Low-fat		High-fat	
	Fibre diets	Fibre-free diets	Fibre diets	Fibre-free diets
Basal diet^1^	409.2	409.2	409.2	409.2
Lard	-	-	230	230
Cholesterol	-	-	20	20
Dietary fibre†	87.9–100	-	87.9–100	-
Wheat starch¥	490.8–502.9	590.8	240.8–252.9	340.8
**E%**				
Fat	11.5–11.6	10.9	50.7–50.9	48.7
Protein Car	21.1-21.4	20.0	21.1–21.4	16.0
Carbohydrate	63.1–63.4	69.1	29.3	35.3
Dietary fibre	3.9–4.0	-	3.1	-

1 Containing (g/kg, dwb): 200 casein (Sigma Aldrich, St. Louis, MO, USA), 50 rapeseed oil (Zeta, Stockholm, Sweden), 1.2 DL-methionine (Sigma Aldrich, St. Louis, MO, USA), 100 sucrose (Nordic sugar, Copenhagen, Denmark), 8 vitamin mixture§, 2 choline chloride (Sigma Aldrich, St. Louis, MO, USA), 48 mineral mixture‡.

† Corresponding to the content of dietary fibre in pectin and guar gum which was 800 g/kg (dwb) and 910 g/kg (dwb), respectively.

§ Containing (g/kg): 0.62 menadione, 2.5 thiamin hydrochloride, 2.5 riboflavin, 1.25 pyridoxine hydrochloride, 6.25 calcium pantothenate, 6.25 nicotinic acid, 0.25 folic acid, 12.5 inositol, 1.25 p-aminobenzoic acid, 0.05 biotin, 0.00375 cyanocobalamin, 0.187 retinol palmitate, 0.00613 calciferol, 25 d-α-tocopheryl acetate, 941.25 maize starch (Lantmännen, Stockholm, Sweden).

‡ Containing (g/kg): 0.37 CuSO_4_·5H_2_O, 1.4 ZnSO_4_·7H_2_O, 332.1 KH_2_PO_4_, 171.8 NaH_2_PO_4_·2H_2_O, 324.4 CaCO_3_, 0.068 KI, 57.2 MgSO_4_, 7.7 FeSO_4_·7H_2_O, 3.4 MnSO_4_·H_2_O, 0.02 CoCl·6H_2_O, 101.7 NaCl, 0.019 chromium(III)chloride and 0.011 sodium selenate.

¥ Norfoods Sweden AB, Malmö, Sweden, varied according to the dietary fibre content of the test materials.

### Animals and diets

**Figure 1:**
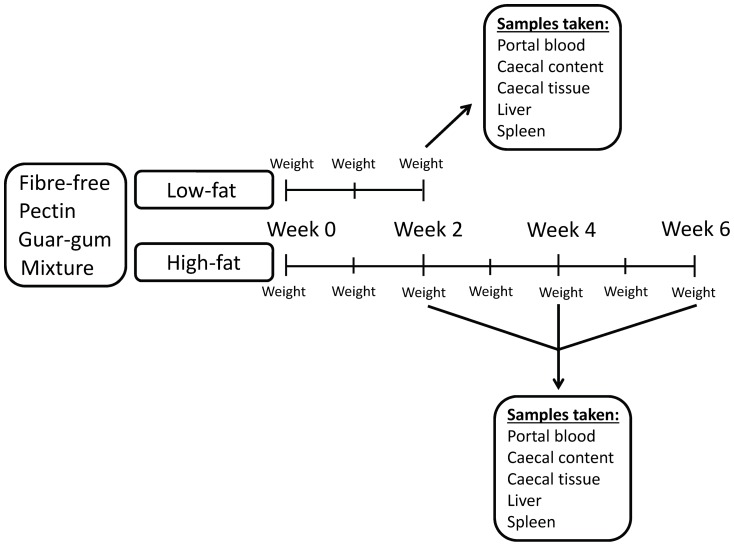
Study design. Schematic illustration of the study design.

Male Wistar rats (Scanbur AB, Sollentuna, Sweden), with an initial weight of 129 g (SE 0.9), were randomly divided into sixteen groups of seven (one group per cage). The rats were housed in a room maintained at 22°C, with a 12 h light-dark cycle. Three test diets containing pectin, guar gum or a mixture of pectin and guar gum (mixture), and a fibre-free control diet, were prepared and given to the rats. The LFD contained 50 g fat per kg dry weight basis (dwb), while the HFD contained 280 g fat per kg (dwb) (Table 1). The design of the study resulted in eight diets, but since the experiment on HFD lasted for 6 weeks instead of 2 weeks and invasive samples were taken every second week, the experiment started with 16 groups (Figure 1). The feed intake was restricted to 12 g dry matter per rat and day (84 g per group and day) for the first three weeks of the experiment, and then to 20 g dry matter per rat and day (140 g per group and day). Water was given *ad libitum*. The dietary fibre content of the diets was 80 g/kg diet (dwb). Wheat starch was used to fill up the carbohydrate concentration. The wheat starch used has been shown to be completely digested, and forms little to no SCFAs [36]. The protocol for the animal experiment was approved by the Ethics Committee for Animal Studies at Lund University (application number: M 30-09). The rats appeared healthy and active through the experimental period and the diets were well tolerated.

After 5 d of acclimatizing, an experimental period of 2, 4 and 6 weeks followed. The experiment on rats fed LFD lasted for 2 weeks, while the experiment on rats fed HFD persisted for 2, 4 and 6 weeks. The weight of the animals was registered every week. At the end of the experimental period, the animals were anaesthetized by subcutaneous injection of a mixture (1∶1∶2) of Hypnorm (fentanyl citrate 0.315 mg/ml and fluanisone 10 mg/l) (Division of Janssen-Cilag Ltd, Janssen Phamaceutica, Beerse, Belgium), Dormicum (midazolam 5 mg/ml) (F. Hoffman-La Roche AG, Basel, Switzerland) and water at a dose of 0.15 ml/100 g body weight. Blood samples (serum and plasma) were collected from the portal vein and placed in plasma tubes containing EDTA (K2E 3.6 mg, Plus Blood Collection Tubes, BD, Plymouth, UK) and serum tubes (SST™ Advance, Plus Blood Collection Tubes, BD, Plymouth, UK). After blood has been collected from the portal vein, the rats were euthanized through an incision in the heart. The samples were centrifuged and stored at −40°C until the analysis of SCFAs, succinic acid, cytokines and cholesterol. The caecum was removed, weighed with and without its content, and the pH of the content was measured, before being stored at −40°C for analysis of SCFAs, lactic and succinic acids. A small part of the caecum content was weighed and collected in sterile tubes containing freezing medium (water, glycerol [98%], MgSO_4_-7H_2_O, Na-Citrate, KH_2_PO_4_, K_2_HPO_4_) and immediately frozen in liquid nitrogen for analysis of the microbiota composition. The spleen and the liver were weighed and the liver was frozen at −20°C for analysis of cholesterol, triglycerides and fat content.

### Analyses

#### Fat content of the liver

Before analysis of fat content, the livers were lyophilized and mortared. The fat content was analysed using the SBR (Schmidt-Bondzynski-Ratzlaff) method. The liver samples were digested in 7.7 M HCl (Merck, Darmstadt, Germany) for 60 min at 75°C before being washed and extracted with ethanol (Kemetyl, Haninge, Sweden), diethylether (Merck, Darmstadt, Germany) and petroleumbensin (Merck, Darmstadt, Germany) for 30 min. The extracts were transferred to a clean beaker, washed twice with 1∶1 diethylether∶petroleumbensin, and allowed to settle for 30 min before the extracts were transferred to a beaker, air-dried and weighed.

#### Cholesterol and triglycerides in liver and blood

Lyophilized and mortared liver tissues were analysed for cholesterol and triglycerides. The lipids were extracted and washed with a 3∶2 mixture of hexane (Sigma Aldrich, St. Louis, USA) and isopropanol (Merck, Darmstadt, Germany) containing 0.005% (v/w) BHT (2,6-Di-Tert-Butyl-4-Metylphenol) (Merck, Munich, Germany) on an orbital shaker followed by centrifugation, and the extracts were then transferred into a clean tube. This procedure was repeated four times. The lipid extracts were dried under N_2_ flow at room temperature and re-dissolved in isopropanol+1% (v/v) Trition X100 (Sigma Aldrich, St. Louis, MO, USA). Total liver cholesterol and triglycerides and plasma cholesterol were determined spectrophotometrically using Infinity™ Cholesterol and Infinity™ Triglycerides reagent and Cholesterol and Triglycerides Standard (Thermo Scientific, Middletown, VA, USA).

#### SCFAs and succinic acid in serum

After centrifugation of the blood, the serum was transferred to a clean tube and analysed with regard to SCFAs (acetic, propionic, iso-butyric, butyric, iso-valeric and valeric acids) using GLC [37]. Water and 2-ethylbutyric acid (internal standard) were added to the serum samples and the SCFAs were protonated with hydrochloric acid (HCl). A hollow fibre, for supported liquid membrane extraction, was immersed in the serum solution to extract the SCFAs. After extraction, the SCFAs were flushed from the fibre lumen before being injected onto a fused-silica capillary column (DB-FFAP 125-3237; J&W Scientific, Agilent Technologies Inc., Folsom, CA, USA). GC ChemStation software (Agilent Technologies Inc., Wilmington, DE, USA) was used for the analysis.

Succinic acid was analysed by mixing 400 µl of serum with 100 µl of 10% (v/v) sulphosalicylic acid to precipitate high-molecular-weight proteins. The samples were vortexed for 30 sec and then centrifuged for 30 min, and the supernatant was filtered through PolyTetraFluoroEthylene (PTFE) Syringe Filters (Pore Size: 0.45 µm, Diameter: 13 mm) (Skandinaviska Genetec AB, Västra Frölunda, Sweden) before quantification with ion-exclusion chromatography (MIC-2 Advanced modular IC) (Metrohm AG, Herisau, Switzerland). The system comprised a serial double-piston high-pressure pumping unit (818 IC), a two-channel peristaltic pump with the Metrohm Suppressor Module, a separation centre (820 IC), a conductivity detector (819 IC), and an interface (830 IC) to connect with a computer. The Metrohm IC Net 2.3 software was used to analyse the chromatograms. Ion-exclusion chromatography with inverse suppression and conductivity detection was used to detect the succinic acid peak. Samples were injected via a 20 µl loop and eluted at a flow rate of 0.6 ml per min and at a pressure of 3.0 Mpa. The column used was Metrosep organic acids analytical column (6.1005.210, 250 mm×7.8 mm, particle size of 10 µm, with polystyrene-divinylbenzene copolymer packing material functionalized with sulphonic acid groups). The eluent solution was 0.5 mM sulphuric acid, which was degassed by nitrogen before use. The solution of 50 mM LiCl and water was pumped at the same speed, which regenerated the suppressor system. Running temperature was 70°C and the running time for each analysis was 25 min. The conductivity detector was operated in the positive mode at a full scale of 10.0 µS/cm. Standard solutions were made with serial dilutions, resulting in five different concentrations, and analysed using the same method as the serum samples (see above). A linear equation was produced, using the peak area and the different concentrations. Then the equation was used to calculate the concentration of succinic acid in the samples.

#### SCFAs and lactic and succinic acids in the caecal content of rats

The SCFAs (acetic, propionic, iso-butyric, butyric, iso-valeric and valeric acids) in the caecal content were analysed using a GLC method [38]. The caecal content was homogenized for 1 min with an Ultra Turrax® T25 basic (IKA®-Werke, Staufen, Germany) after the addition of hydrochloric acid and 2-ethylbutyric acid (internal standard). Hydrochloric acid was added to protonate the SCFAs. The samples were then centrifuged (MSE Super Minor, Hugo Tillquist AB, Solna, Sweden) before injection onto a fused-silica capillary column (see above). The remaining supernatant was frozen for later analysis of lactic and succinic acids with ion-chromatography. For analysis, the samples were thawed and centrifuged again and the supernatant was filtered through a PTFE Syringe Filter and analysed for lactic and succinic acids, as described above.

#### Multiple cytokine assays

A Milliplex micro-beads array system was used to simultaneously measure serum levels of the following eight cytokines: interleukin (IL)-1α, IL-1β, IL-6, IL-10, IL-18, MCP-1, IFNγ, and TNFα. The assay was conducted according to the manufacturer's instructions, using Milliplex™ MAP rat cytokine kit assay technology (Millipore Corp., Billerica, MA, USA). The antibody specific to each cytokine was coupled to microspheres that were uniquely labelled with a fluorescent dye. The microspheres were incubated with standards, controls and samples in a 96-well filter plate overnight at 4°C. After incubation, the plate was washed to remove excess reagent, and detection antibodies, one for each of the eight cytokines, were added to the vials. After 2 h incubation at room temperature, streptavidin-phycoerythrin was added for a further 30 min. A final wash step was included before the beads were resuspended in buffer and read on the Luminex 200 instrument (Luminex Corporation, USA) to determine the level of the cytokine of interest. All specimens received were tested in replicate wells. Milliplex™ Analyst v. 3.4 (Millipore) was used for the evaluation of the results.

#### Terminal Restriction Fragment Length Polymorphism (T-RFLP) analysis

The T-RFLP analysis was performed on three groups fed HFD (fibre-free, pectin and guar gum diets) for 6 weeks. The reason for this was that the caecal microbiota is probably most affected and adapted to each diet after a longer experimental period.

The caecal contents were thawed on ice and centrifuged at 10 000 rpm for 5 min. The supernatant was discarded and the pellet was resuspended in 1–4 ml of 1XPBS (Oxoid, Basingstoke, UK) depending on the weight of the caecal content. Total DNA was extracted using EZ1 DNA tissue kit (Qiagen, Hilden, Germany) according to the manufacturer's instructions. The 16S rRNA genes were amplified using a fluorescently labelled forward primer ENV1 (5′-FAM-AGAGTTTGATIITGGCTCAG-3′) and an unlabelled reverse primer ENV2 (5′-CGGITACCTTGTTACGACTT-3′). The PCR reaction was prepared in a total volume of 25 µl containing 0.4 µM of FAM-ENV1 primer and 0.2 µM of primer ENV2, 2.5 µl of 10 x PCR reaction buffer (500 mM Tris-HCl, 100 mM KCl, 50 mM (NH_4_)_2_SO_4_, 20 mM MgCl_2_, pH 8.3), 0.2 mM of each deoxyribonucleotide triphosphate, 2.5 U of FastStart Taq DNA polymerase (Roche Diagnostics, Mannheim, Germany), and 2 µl of template DNA. The PCR was performed in an Eppendorf MasterCycler (Eppendorf, Hamburg, Germany) using the following programme: 95°C for 3 min, 94°C for 3 min, followed by 30 cycles of 94°C for 1 min, 50°C for 45 sec, and 72°C for 2 min. Finally, an additional extension at 72°C for 7 min was done. Triplicate reactions were carried out for each sample and a negative control was included in all PCR runs. After the amplification, the 16S rDNA amplicons were verified by Agarose Gel Electrophoresis. The amplicons of each sample were then pooled and purified by MinElute PCR Purification Kit (Qiagen, Hilden, Germany) according to the manufacturer's instructions. The purified DNA concentration was measured by Nanodrop ND-1000 (Saveen Werner, Limhamn, Sweden). Then 200 ng of the purified DNA were digested with 10 U of the restriction endonucleases *Msp*I (Fermentas Life Science, Burlington, Canada) in a total volume of 10 µl for 5 h at 37°C followed by 20 min enzyme inactivation at 65°C. After digestion, aliquots of the products were diluted 5 times with sterile water. Diluted samples were then transferred to sterile 96-well plates (Becton Dickinson, Franklin Lakes, NJ, USA) for T-RFLP analysis on 3130×l Genetic Analyzer (Applied Biosystems, Warrington, UK). In all samples a DNA size marker GeneScan™ LIZ 600 (Applied Biosystem) was included. Fragment sizes, peak height and peak area were analysed with Genemapper® software version 4.0 (Applied Biosystems). The relative area for each terminal restriction fragment (T-RF) was calculated by dividing the peak area of each T-RF with the total peak area for a sample.

### Statistical evaluation

The experimental design was randomized. The eight test diets contained three fibre groups, pectin, guar gum, or a mixture, and one fibre-free group. These four test diets were administered either as LFD or HFD, resulting in a total of eight diets. The experiments with LFD lasted for 2 weeks, while the experiment with HFD continued for 2, 4 or 6 weeks.

**Figure 2:**
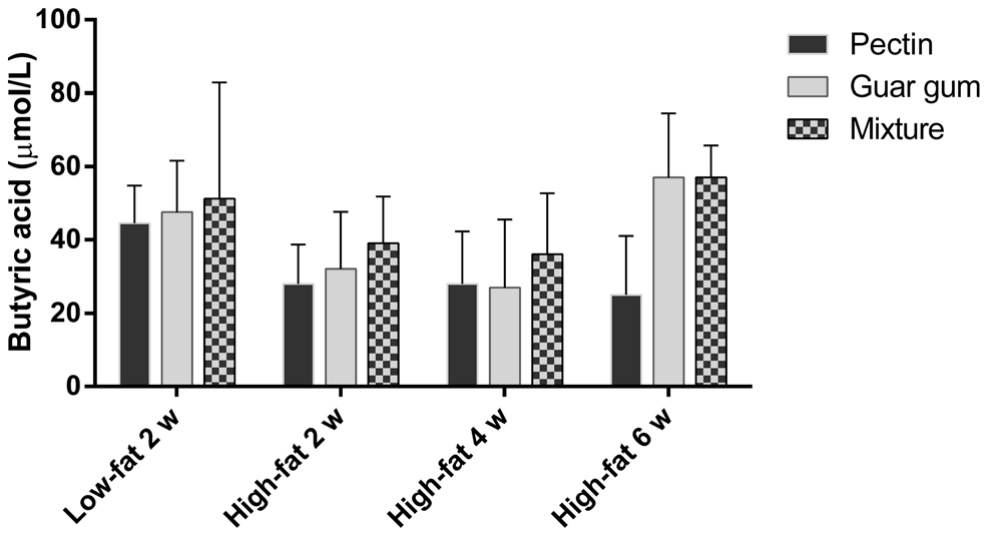
Serum concentration of butyric acid. Serum concentration (µmol/L) of butyric acid in rats fed the three dietary fibre diets for 2, 4 and 6 weeks (means±SEM, n = 7, with exception of groups pectin and fibre-free diets for 4 and 6 weeks, respectively, n = 6).

**Figure 3:**
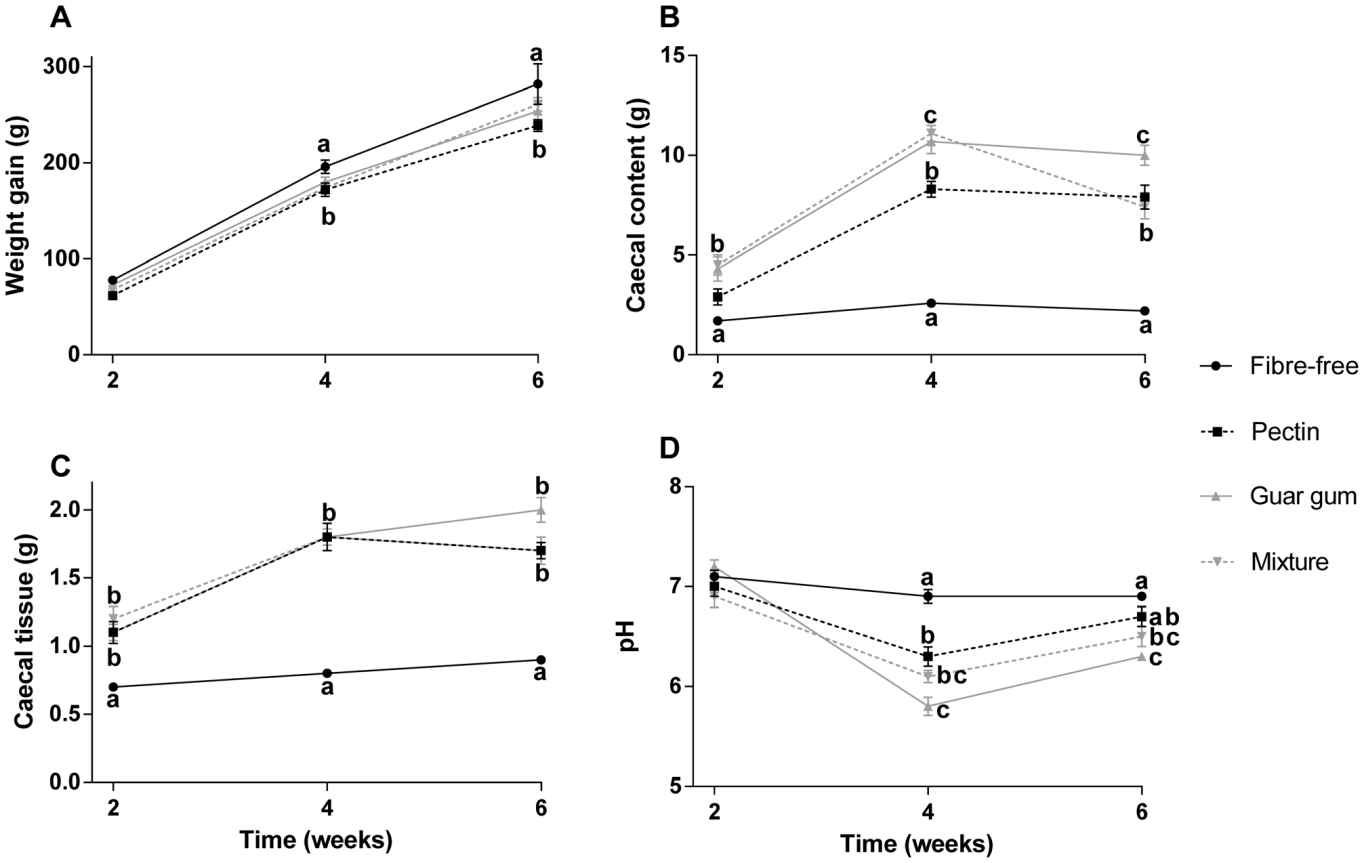
Weight gain, caecal weight and pH. A: Weight gain (g), B: weight of caecal content (g), C: caecal tissue weight (g) and D: pH in rats fed the four HFD for 2, 4 and 6 weeks (means ± SEM, n = 7). Values with different letters are significantly different, p<0.05.

**Figure 4:**
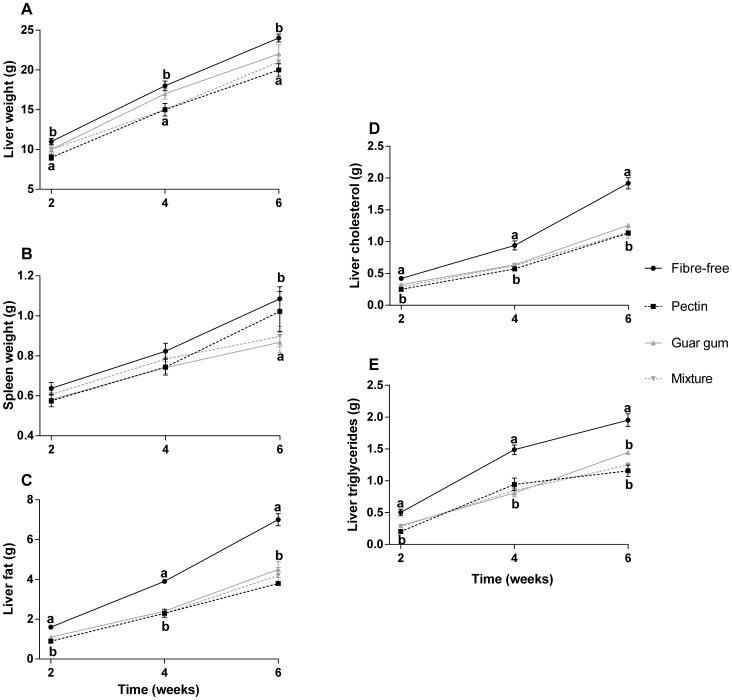
Organ weight and analytical markers. A: liver weight (g), B: spleen weight (g) C: liver fat content (g), D: liver cholesterol (g) and E: liver triglyceride (g) in rats fed the four HFD for 2, 4 and 6 weeks (means ± SEM, n = 7). Values with different letters are significantly different, p<0.05.

**Figure 5:**
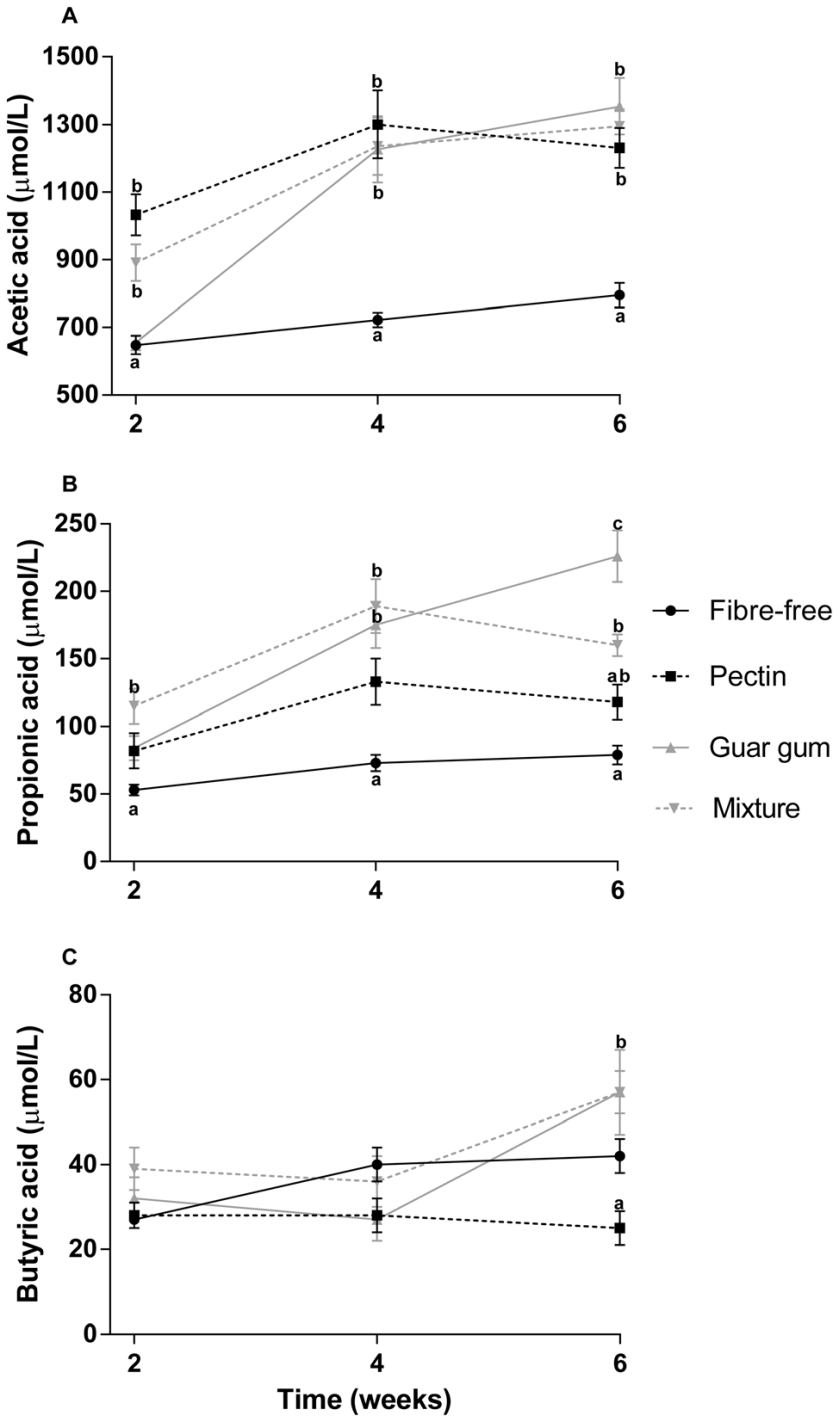
Serum concentration of SCFAs. Serum concentration (µmol/L) of A: acetic acid, B: propionic acid and C: butyric acid in rats fed the four HFD for 2, 4 and 6 weeks (means ± SEM, n = 7, with exception of groups pectin and fibre-free diets for 4 and 6 weeks, respectively, n = 6). Values with different letters are significantly different, p<0.05.

**Figure 6:**
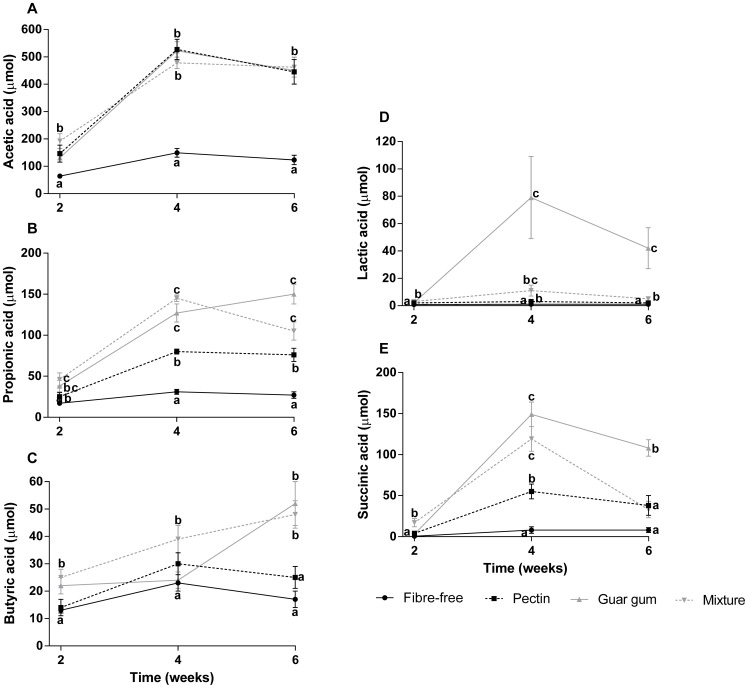
Caecal pools of CAs. Caecal pools (µmol) of A: acetic acid, B: propionic acid, C: butyric acid, D: lactic acid and E: succinic acid in rats fed the four HFD for 2, 4 and 6 weeks (means ± SEM, n = 7, with exception of group guar gum diet for 6 weeks, respectively, n = 6). Values with different letters are significantly different, p<0.05.

**Figure 7:**
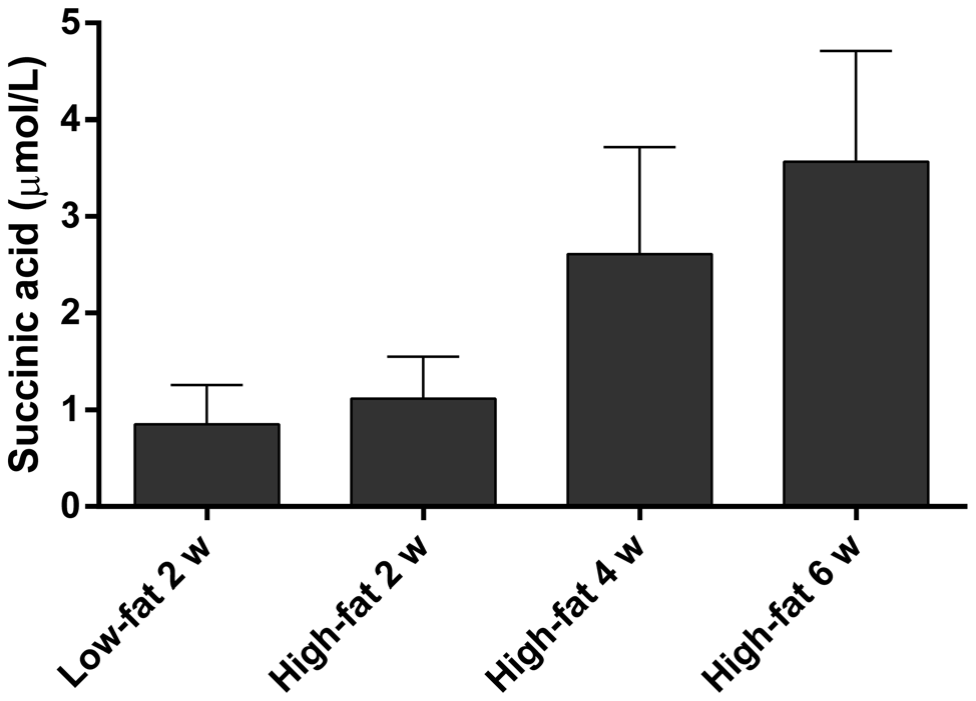
Serum concentration of succinic acid. Concentration of succinic acid (µmol/L) in serum from rats fed the fibre-free LFD for 2 w and HFD for 2, 4 and 6 weeks (means ± SEM, n = 7, 6, 4 and 6 for LFD 2 w, HFD 2 w, 4 w and 6 w, respectively).

**Figure 8:**
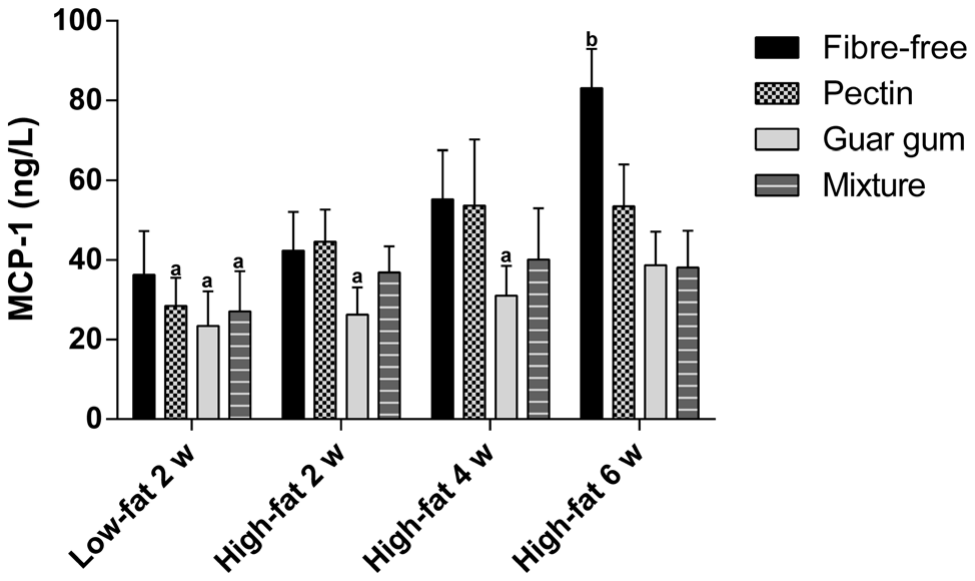
Serum concentration of MCP-1. Concentration (ng/L) of MCP-1 in portal serum in rats fed the four LFD for 2 weeks and HFD for 2, 4 and 6 weeks (means ± SEM, n = 7, with exceptions of groups fed pectin, guar gum and mixture with low-fat content for 2 weeks, fibre-free with high-fat content for 4 weeks and fibre-free and pectin with high-fat content for 6 weeks, n = 6). Values with different letters are significantly different, p<0.05.

**Figure 9:**
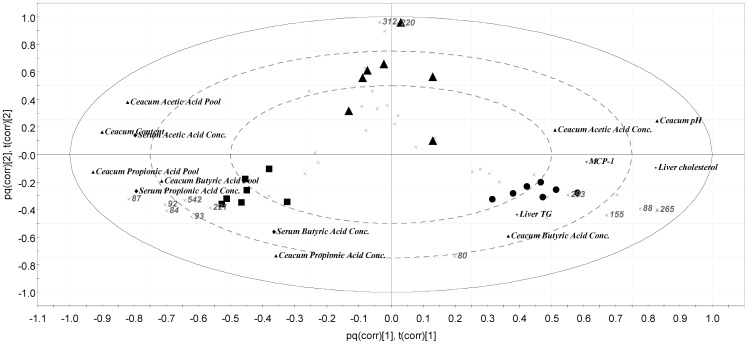
Grouping of the caecal microbiota. Loading Bi plot of the grouping of the caecal microbiota and analytical markers in rats fed fibre-free (dots), pectin (triangles) and guar gum (squares) diets with high-fat content for 6 weeks (n = 7).

**Figure 10:**
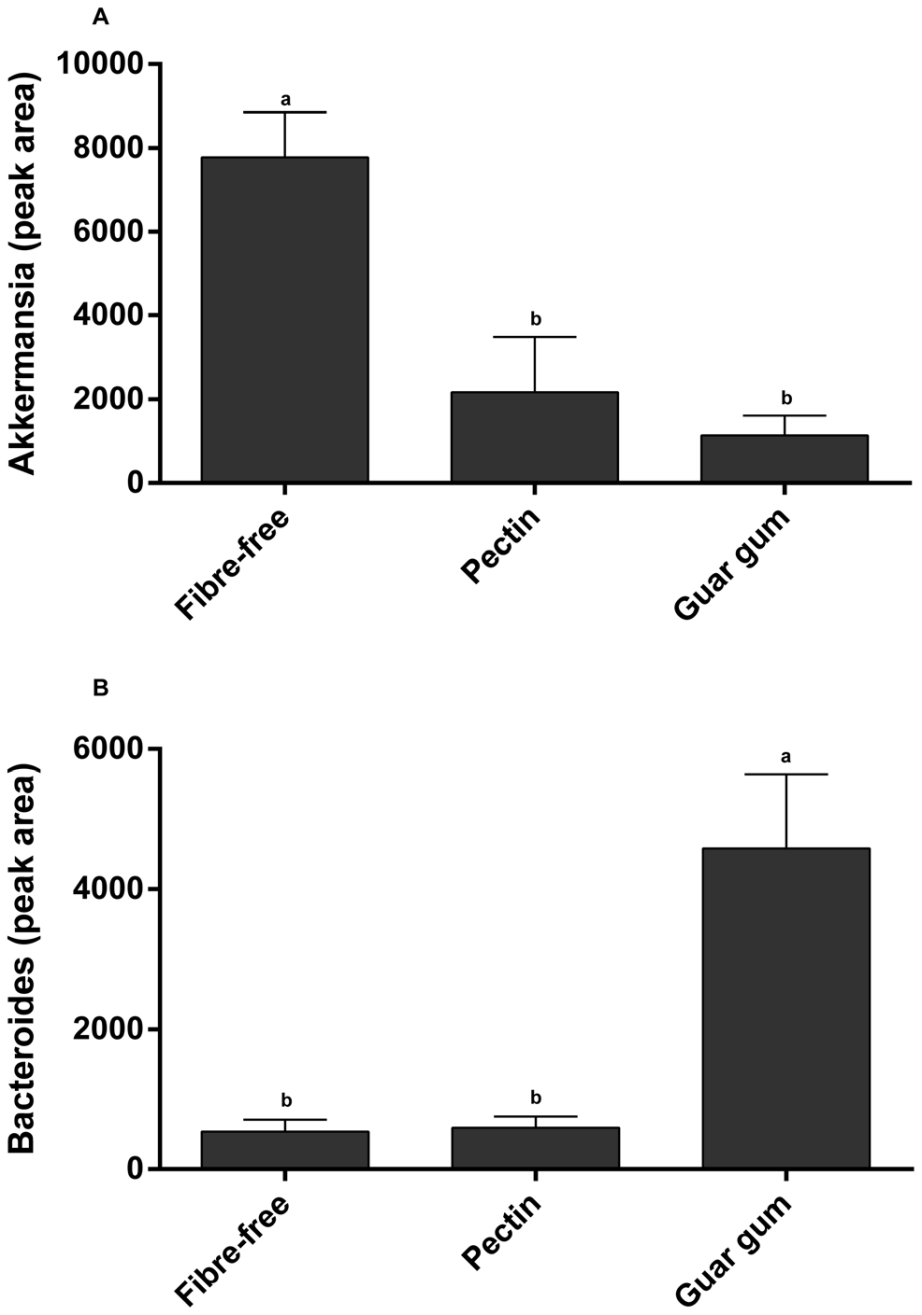
*Akkermansia* and *Bacteroides*. Peak area of T-RFLP peaks for *Akkermansia* and *Bacteroides* in rats fed the fibre-free, pectin and guar gum diets for 6 weeks (n = 7). Values with different letters are significantly different, p<0.05.

Two-way ANOVA was used to determine the effects of dietary fibre (Fibre), fat content (Fat) and their interaction (Fibre×Fat) for the groups fed LFD and HFD for 2 weeks (Tables 2, 3, 4, 5; p<0.05). Data from rats fed the different test diets with the same length of experimental period were compared with regard to weight gain, caecal content, tissue weight and pH, liver and spleen weight, liver cholesterol, triglyceride and fat and plasma cholesterol, and SCFAs and lactic and succinic acids (Tables 2, 3, 4, 5, Figures 2, 3, 4, 5, 6). In addition, all the groups, regardless of the length of the experimental period, were compared with regard to concentration of succinic acids in serum and inflammatory cytokines (Figure 7 and 8). This was done with one-way ANOVA (p<0.05). Length of experimental period and different analytical markers were correlated using Pearson product moment correlation coefficient. All evaluations, with the exception of the T-RFLP analysis, were performed with Minitab software (release 16). The results were normally distributed, with the exception of results from the T-RFLP analysis, weight gain after 4 weeks, caecum content weight after 2 weeks, liver and spleen weight after 6 weeks, liver cholesterol and triglyceride, triglyceride in plasma for all time-points, which became normally distributed after Box-Coxing Transformation of the dataset (with the exception of T-RFLP). The pH values for the 6 weeks experimental period were compared using Kruskal-Wallis. Shannon's diversity index [39] was not normally distributed and was evaluated with the Kruskal-Wallis test using package “coin” in the R program (version 2.15.1). T-RFLP data together with SCFA data and physiological data were analysed with Orthogonal Partial Least Squares Projections to Latent Structures (OPLS) with SIMCA-P software (version 12.0.1.0, Umetrics, Umeå, Sweden) (Figure 9). The results are shown as means and standard error in Tables 2, 3, 4, 5 and Figures 2, 3, 4, 5, 6,7, 8 and 10.

**Table 2:** Weight gain, weight of caecal content, tissue, liver and spleen (g) and caecal pH in rats fed the four test diets with low-fat or high-fat content for 2 weeks (means ± SEM, n = 7).

Fat	Diet	Weight gain (g)	Caecal content (g)	Caecal tissue (g)	Caecal pH	Liver weight (g)	Spleen weight (g)
**Low**	Fibre-free	59±2	1.4±0.1^a^	0.6±0.02^a^	6.9±0.1^1^	8.3±0.3	0.58±0.04
	Pectin	58±3	3.2±0.3^b^	0.8±0.04^b^	7.0±0.05	7.1±0.5	0.59±0.2
	Guar gum	61±3	3.8±0.3^bc^	1.0±0.04^c^	7.0±0.08	7.6±0.3	0.58±0.03
	Mixture	60±3	2.8±0.3^b^	0.9±0.04^bc^	7.1±0.04	7.1±0.1	0.59±0.02
**High**	Fibre-free	78±4^a^ **	1.7±0.1^a^	0.7±0.03^a^	7.1±0.06	10.6±0.4*	0.64±0.03
	Pectin	62±4^b^	2.9±0.4^ab^	1.1±0.08^b^ *	7.0±0.1	8.6±0.3	0.58±0.03
	Guar gum	73±2	4.3±0.6^b^	1.1±0.06^b^	7.2±0.07	9.5±0.3*	0.58±0.02
	Mixture	68±4	4.5±0.5^b^ *	1.2±0.09^b^ *	6.9±0.1	9.6±0.6***	0.61±0.02

a,b,c Mean values within a column, for different fat levels, with unlike superscripts are significantly different (P<0.05).

Mean values were significantly different from those of rats fed the corresponding low-fat diet: * P<0.05, ** P<0.01, ***P<0.001.

1 n = 5.

**Table 3:** Total amount of fat (g), cholesterol (mg) and triglyceride (mg) in liver and plasma cholesterol concentration (mmol/L) in rats fed the four test diets with low-fat or high-fat content for 2 weeks (means ± SEM, n = 7).

		Fat content	Cholesterol	Triglyceride	Cholesterol
Fat	Diet	Liver (g)	Liver (mg)	Liver (mg)	Plasma (mmol/L)
**Low**	Fibre-free	0.31±0.03^a^	79±3^a^	168±19^a^	2.9±0.1
	Pectin	0.23±0.02^b^	65±6^ab^	128±10^ab^	3.0±0.1
	Guar gum	0.24±0.01^ab^	65±3^ab^	112±5^b^	2.8±0.07
	Mixture	0.23±0.01^b^	63±1^b^	106±2^b^	2.7±0.08
**High**	Fibre-free	1.59±0.1^a^***	422±26^a^***	503±52^a^***	4.0±0.2^a^***
	Pectin	0.86±0.05^b^***	252±13^b^***	204±12^b^**	3.3±0.1^b^
	Guar gum	1.10±0.1^b^***	327±26^b^***	296±25^b^***	3.4±0.1^b^**
	Mixture	0.90±0.02^b^***	293±17^b^***	282±14^b^***	3.8±0.1^ab^***

a,b Mean values within a column, for different fat levels, with unlike superscripts are significantly different (P<0.05).

Mean values were significantly different from those of rats fed the corresponding low-fat diet: * P<0.05, ** P<0.01, ***P<0.001.

**Table 4:** Serum concentration (µmol/L) of SCFAs in rats fed the four test diets with low-fat or high-fat content for 2 weeks (means ± SEM, n = 7).

Fat	Diet	Acetic acid	Propionic acid	Butyric acid	Minor acids	Total SCFA
**Low**	Fibre-free	723±46^a^	49±7^a^	26±4	78±2^a^	876±59^a^
	Pectin	1111±81^b^	86±9^ab^	45±5	94±7^ab^	1336±97^b^
	Guar gum	723±39^a^	129±20^b^	48±7	126±16^b^	1026±73^a^
	Mixture	766±24^a^	71±11^a^	36±8	93±8^ab^	982±53^a^
**High**	Fibre-free	648±27^a^	53±4^a^	27±2	94±5	823±32^a^
	Pectin	1033±61^b^	82±13^ab^	28±3	83±11	1226±79^b^
	Guar gum	655±22^a^	84±9^ab^	32±5	87±6	859±32^a^
	Mixture	891±54^b^	115±13^b^	39±5	72±4	1116±71^b^

a,b Mean values within a column, for different fat levels, with unlike superscripts are significantly different (P<0.05).

**Table 5:** Caecal pools of SCFAs, lactic and succinic acids (µmol) in rats fed the four test diets with low-fat or high-fat content (means ± SEM, n = 7).

Fat	Diet	Acetic acid	Propionic acid	Butyric acid	Minor acids	Total SCFA	Lactic acid	Succinic acid
**Low**	Fibre-free	77±6^a^	12±2^a^	10±0.7^a^	5±0.8^a^	90±9^a^	0.7±0.1	0.4±0.05^a^
	Pectin	198±24^c^	30±4^bc^	22±2^b^	10±0.8^b^	260±30^b^	1.8±0.4	0.9±0.2^b^
	Guar gum	135±19^bc^	42±8^c^	23±3^b^	11±1^b^	210±31^b^	2±0.4	1±0.1^b^
	Mixture	123±13^ab^	22±3^ab^	22±5^b^	8±1^ab^	176±20^ab^	1±0.3	0.9±0.1^b^
**High**	Fibre-free	64±6^a^	17±1^a^	13±1^a^	7±0.7^ab^	114±9^a^	0.9±0.1^a^	0.6±0.1^a^
	Pectin	146±31^ab^	25±5^b^	14±3^a^	6±0.9^a^	191±39^ab^	2±0.4^ab^	4±2^ab^
	Guar gum	131±15^ab^	38±7^bc^	22±3^ab^	10±1^b^	201±24^ab^	2±0.8^ab^	3±1^ab^
	Mixture	192±27^b^*	46±8^c^*	25±3^b^	9±1^ab^	272±36^b^	3±0.8^b^	17±5^b^*

a,b,c Mean values within a column, for different fat levels, with unlike superscripts are significantly different (P<0.05).

Mean values were significantly different from those of rats fed the corresponding low-fat diet: * P<0.05.

The concentration of each SCFA (µmol/g) and the amount of liver cholesterol and triglyceride (mg/g and g/g) was multiplied by either the caecal content weight and liver weight, to obtain the caecal pool of SCFA (µmol) and the total amount of liver cholesterol and triglyceride (mg and g). P-values close to significance (p≤0.1) were defined as tendency.

## Results

### Low-fat diets (2 weeks)

#### Weight gain, caecal content, tissue weight and pH, liver and spleen weight

Body weight gain was similar for all groups, and thus independent of whether dietary fibre was added to the diets or not (58–61 g) (Table 2). The weight of the caecal content and tissue increased in line with dietary fibre in the diet (p<0.001), while no differences were seen between any groups for the caecal pH. Rats fed the fibre-free diet had a tendency to heavier liver weights (8.3 g) than rats fed pectin and the mixture (7.1±0.2 g, p<0.1). No difference could be seen for the spleen weights between groups with any of the diets.

#### Liver fat, cholesterol and triglycerides and plasma cholesterol

Rats fed pectin and the mixture had lower content of fat in the liver compared with the fibre-free group (0.23±0.01 g vs. 0.31 g, p<0.05) (Table 3). The total amount of liver cholesterol was lower in rats fed the mixture compared with rats fed the fibre-free diet (63 mg vs. 79 mg, p = 0.0256). Furthermore, it was close to significant for rats fed pectin and guar gum (65±3 mg, p<0.06) compared with the fibre-free group. The addition of guar gum and the mixture decreased the total amount of triglycerides in the liver of rats (112 and 106 mg, respectively, vs. 168 mg, p<0.01). Similar trends could be seen for the concentrations (mg/g) of cholesterol and triglycerides (data not shown). There was no difference in plasma cholesterol concentration between groups.

#### Serum concentration of SCFAs

The concentration of acetic acid in portal serum of the rats was between 723 and 1111 µmol/L, of propionic acid between 49 and 129 µmol/L, and of butyric acid between 26 and 48 µmol/L (Table 4). The minor SCFAs (iso-butyric, valeric and iso-valeric acids) were also present in considerable amounts (78–126 µmol/L). Pectin generated the highest concentration of acetic acid (p<0.001) and guar gum the highest propionic acid concentration (p<0.05). The group fed guar gum had also higher concentration of the two minor acids, iso-valeric and iso-butyric acids (p<0.05), and a tendency towards a higher concentration of butyric acid (p = 0.06) compared with the fibre-free group.

#### Caecal pools of carboxylic acids

The main SCFA in the caecal pool was acetic acid (77–198 µmol), followed by propionic acid (12–42 µmol) and butyric acid (10–23 µmol) (Table 5). The minor acids were produced in small amounts (5–11 µmol). Pectin generated the highest amount of acetic acid (p = 0.0001), while guar gum gave the highest amount of propionic acid compared with the fibre-free group (p = 0.045). All the fibre groups gave similar amounts of butyric and succinic acids, which were small concerning succinic acid, but all values with the fibre groups were higher than those seen in the fibre-free group (p<0.05). No difference between groups was seen for lactic acid.

### Effect of high-fat diets (2 weeks)

#### Weight gain, caecal content, tissue weight and pH, liver and spleen weight

The average body weight was higher in rats fed the HFD than rats fed the LFD (mean 70±2 g vs. mean 60±1 g, p<0.001) (Table 2). The body weight gain was highest in the rats fed fibre-free diet, 32% higher (78 g vs. 59 g, p = 0.0047) for the HFD compared with corresponding LFD. A tendency towards a lower weight gain was seen when dietary fibre was added to HFD (78 g vs. mean 68±2 g for rats fed dietary fibre, p = 0.057, corresponding to a 7–20% lower body weight gain). In this respect, pectin had the most pronounced effect (p = 0.0218, corresponding to 7% higher weight with the HFD than with corresponding LFD).

The average weight of the caecal content and tissue was higher in groups fed HFD than LFD (mean 3.3±0.3 g vs. mean 2.8±0.2 g for caecal content and mean 1.0±0.05 g vs. mean 0.8±0.05 g for caecal tissue, p<0.001). The weights were also higher with dietary fibre in the diets than without any fibre (p<0.001). When comparing the individual groups, only the mixture gave a higher caecal content weight with HFD than with corresponding LFD (4.5 g vs. 2.8 g, p = 0.039). Caecal tissue weight was higher in rats fed pectin and the mixture compared with corresponding LFD (1.1 g vs. 0.8 g, p = 0.016 for pectin and 1.2 vs. 0.9 g, p = 0.032 for the mixture). No differences in caecal pH were observed between LFD and HFD.

The average liver weight was higher in groups fed the HFD compared with LFD (mean 9.6±0.2 g vs. mean 7.5±0.2 g, p<0.001). Pectin was most effective in lowering the liver weight in the groups fed HFD (from 10.6 to 8.6 g, p = 0.011). When comparing rats fed corresponding LFD and HFD, all groups with the exception of pectin had significantly heavier liver weight with HFD (p = 0.0017 [fibre-free], p = 0.0106 [guar gum] and p = 0.0007 [mixture]) compared with LFD. The appearance of the liver tissue was also affected by the fat content, and the colour was light and yellow with HFD, while rats fed LFD had dark red livers. No difference could be seen for the spleen weights with any of the HFD, and not compared with LFD either.

#### Liver fat, cholesterol and triglycerides and plasma cholesterol

The fat and cholesterol content of the liver was four to five times higher with HFD than with LFD for all groups (p<0.001), even though the values were significantly lower with dietary fibre in the diet (Table 3). Consequently, the addition of dietary fibre resulted in 40±3% lower liver fat (p<0.001), while the amount of cholesterol decreased by 31±3% (p<0.02).

Similar results could be seen in liver triglycerides. The total amount of triglycerides in the liver was higher in the rats fed HFD compared with the LFD (p<0.01). This was valid for all groups, but the difference was highest between the fibre-free groups, i.e. the addition of dietary fibre reduced the effect of fat in the diet (p<0.001).

Plasma cholesterol concentrations were higher with HFD than with LFD (p = 0.005), in all groups except those fed pectin. Comparing the different HFD, groups fed either pectin or guar gum had lower plasma concentrations of cholesterol than the group fed a fibre-free diet (p<0.03).

#### Serum concentrations of SCFAs

Similar concentrations of acetic acid could be seen with HFD as for LFD (Table 4). Rats fed pectin had the highest concentration of acetic acid, while rats fed the mixture had the highest concentration of propionic acid. The butyric acid concentration was lower with HFD than with LFD (p = 0.046) (Figure 2). For rats fed guar gum, there was a redistribution from propionic to acetic acid in rats fed HFD (from ratio 5.6 in LFD to 7.8 in HFD). Otherwise, minor difference in serum concentrations for acetic and propionic acid could be seen between LFD and HFD.

#### Caecal pools of carboxylic acids

The mixture generated the highest pool of acetic, propionic and butyric acids in rats fed HFD (p<0.05) (Table 5). The concentration of butyric acid was reduced with the higher level of fat in the diet (p = 0.002) (data not shown). The total amounts of the intermediary carboxylic acids, lactic and succinic acids, were higher in rats fed the mixture and HFD than rats fed corresponding LFD (p = 0.001 and p = 0.0071, respectively). Succinic acid was higher in groups fed HFD than in those fed LFD, especially with the mixture (p<0.001).

### Effect of time (2, 4 and 6 weeks on high-fat diets)

#### Weight gain, caecal content, tissue weight and pH, liver and spleen weight

Rats given dietary fibre had a lower weight gain than those given a fibre-free diet throughout the experimental period, and pectin had most pronounced effects (62 g vs. 78 g for the fibre-free diet, p = 0.0218 after 2 weeks, 172 g vs. 196 g for the fibre-free diet after 4 weeks, p = 0.0314 and 239 g vs. 282 g for the fibre-free diet after 6 weeks, p = 0.0036) (Figure 3).

Both the caecal content and tissue weight were quite similar for the groups fed the fibre-free diet throughout the experiment, but lower compared with the other groups. The caecal content weight was 1.7 g vs. mean 3.9±0.3 g for the fibre groups after 2 weeks; 2.6 g vs. mean 10.0±0.4 g for the fibre groups after 4 weeks; and 2.2 g vs. mean 8.4±0.4 g for the fibre groups after 6 weeks (p<0.01). Similar results could be seen for the tissue weight (p<0.001) when compared with the dietary fibre diets. For groups fed dietary fibre, the caecal content and tissue weights were considerably higher after 4 weeks than after 2 weeks, while the weights remained unchanged or even decreased after 6 weeks. Groups fed pectin generally had a lower caecal content weight than the other dietary fibre groups but, at 6 weeks, caecal content in rats fed the mixture also weighed less than rats fed guar gum (8.3 g vs. 10.7 and 11.1 g for guar gum [p = 0.0052], and the mixture [p = 0.0009], respectively at 4 weeks and 7.9 vs. 10.0 for guar gum at 6 weeks, [p = 0.0366]).

No difference in pH between groups was seen after 2 weeks, but at 4 and 6 weeks the fibre-free group had a higher pH than the other groups (6.9 vs. mean 6.1±0.06 at 4 weeks, p<0.001 and 6.9 vs. mean 6.5±0.06 at 6 weeks, p<0.001). Groups fed guar gum gave the lowest pH, which was significantly lower compared with pectin (5.8 vs. 6.3, p = 0.0046 after 4 weeks).

The liver weights increased with the length of the experiment (Figure 4). The fibre-free groups had higher liver weights than groups fed pectin (10.6 g vs. 8.6 g after 2 weeks [p = 0.0106], 17.9 g vs. 15.5 g after 4 weeks [p = 0.0437], and 23.7 g vs. 19.7 g after 6 weeks [p = 0.0086]). A clear correlation was seen between the length of the experimental time and the liver weights (correlation factor 0.917 and p<0.001).

No difference was seen in the spleen weight after 2 or 4 weeks, but after 6 weeks the fibre-free group had a higher spleen weight than the guar gum group (1.09 g vs. 0.87 g, p = 0.043).

#### Liver fat, cholesterol and triglycerides and plasma cholesterol

The length of the experiment had an influence on liver cholesterol, triglycerides and fat content, and the amount increased with time (correlation factor 0.86, 0.82 and 0.84, respectively, p<0.001). The addition of dietary fibre counteracted this to some extent (Figure 4).

The fibre-free group had more fat in the liver than the groups fed the various fibre diets (p<0.005, p<0.001 and p<0.001, respectively after 2, 4 and 6 weeks). Similar results were seen with cholesterol and triglycerides. The fibre-free groups had the highest amount of liver cholesterol at the end of each experimental period (0.4 g vs. mean 0.3 ± 0.01 g [p<0.05], 0.9 g vs. mean 0.6 ± 0.02 g [p<0.001] and 1.9 g vs. mean 1.2 ± 0.05 g [p<0.001], after 2, 4 and 6 weeks, respectively). No difference was seen between the different fibre groups. The amount of triglyceride was highest in the groups fed the fibre-free diet, 0.5 g vs. mean 0.3 ± 0.02 g (p<0.001) after 2 weeks, 1.5 g vs. mean 0.9 ± 0.07 g (p<0.001) after 4 weeks and 2.0 g vs. mean 1.3 ± 0.04 g for after 6 weeks (p<0.05).

No correlation could be found between plasma cholesterol concentrations and a longer experimental period (data not shown). However, after 2 weeks, pectin and guar gum had lower concentrations than the group fed the fibre-free diet (3.3 and 3.4 vs. 4.0 mmol/L, p<0.05) and, after 4 weeks, rats fed the mixture had the lowest concentrations (2.9 vs. 3.8 mmol/L for the fibre-free group, p = 0.0026). Remarkably, the group fed guar gum had the highest plasma cholesterol concentrations after 6 weeks (4.2 vs. 3.2 ± 0.09 mmol/L for the other groups, p<0.05).

#### Serum concentration of SCFAs

The concentrations of acetic and propionic acids were considerably higher at week 4 than week 2, while the concentrations at week 6 were similar to the ones at week 4 (Figure 5). Butyric acid increased for all groups except pectin at week 6. A correlation was found between the length of the experiment and the concentration of acetic and propionic acids (correlation factors 0.49 and 0.42, respectively, p<0.001). The fibre groups had significantly higher concentration of acetic acid than the fibre-free groups at all times (p<0.001). The serum concentration of succinic acid for the groups fed the fibre-free diets tended to increase with a longer experimental time (p = 0.063) (Figure 7). The concentration of butyric acid declined slowly with time, with the exception of the group fed guar gum and the mixture, which increased considerably after 6 weeks. If these values are excluded, the decline of butyric acid over time was significant (correlation factor −0.41 and p<0.001) (Figure 2).

#### Caecal formation and pool of carboxylic acids

The caecal pool of acetic, propionic, butyric and succinic acids increased with the length of the experimental period (correlation factors 0.50, 0.42, 0.35 and 0.51, respectively, and p<0.001) (Figure 6). There seems to be an optimum after 4 weeks, except for butyric acid concentrations in rats fed guar gum.

### Serum concentration of MCP-1

The lowest concentration of MCP-1 was seen in the group fed guar gum in LFD (23.5 ng/L) (Figure 8). The guar gum groups also tended to have the lowest concentration of MCP-1 after each experimental period. The fibre-free group fed HFD for 6 weeks had a higher concentration (83.1 ng/L) compared with the fibre groups fed LFD for 2 weeks and guar gum groups fed HFD for 2 and 4 weeks (p<0.05). A clear correlation for increased concentration of MCP-1 with HFD and longer experimental period was seen for the fibre-free groups (correlation factor 0.56 and p = 0.013), and dietary fibre counteracted this effect to some extent. The other seven cytokines (IL-1α, IL-1β, IL-6, IL-10, IL-18, IFNγ, and TNFα) could not be detected in sufficient quantities to give reliable values.

### Microbiota composition (6 weeks) and principal component analysis

The T-RFLP profiles for the individual animals in the three high-fat groups, given pectin, guar gum and the fibre-free diet for 6 weeks, were compared by Principal Component (PC) Analysis. The three groups formed separate clusters (Figure 9), 26.8% of the variance was explained by the PC-one and 32.2% by PC-two. Each group resulted in specific microbiota which was then linked to different analytical parameters by Orthogonal Projections to Latent Structures (OPLS). The group fed guar gum correlated with serum and caecal butyric acid, and caecal propionic acid and the bacterial genus *Bacteroides*. The rats fed guar gum had high abundance of bacteria groups represented by T-RF 87, 84, 93, 542 and 221, and those were correlated to the above mentioned analytical markers. The abundance of *Bacteroides* was higher in the group fed guar gum than pectin or the fibre-free diet (p<0.05) (Figure 10). On the other hand, the group fed the fibre-free diet associated well with parameters known to have negative effects such as liver cholesterol and triglyceride, MCP-1, in addition to a correlation to the bacterial genus *Akkermansia*. The abundance of *Akkermansia* was higher in the group fed the fibre-free diet when compared with the groups fed pectin or guar gum (p<0.005) (Figure 10). The group fed pectin was linked to caecal acetic acid concentrations and had greater individual variance within the group in comparison with the other groups. No difference was seen concerning the caecal microbial diversity. However, the fibre-free group and the pectin groups had similar diversity; the diversity increased to some extent with the addition of guar gum.

## Discussion

Overweight, obesity and NAFLD are increasing rapidly, and consequently the rate of related public health diseases, like type 2 diabetes and cardiovascular disease is increasing as well [2]. These diseases can be associated with the metabolic syndrome and may be prevented by dietary modifications. Dietary fibre plays an interesting role through SCFAs, particularly propionic and butyric acids, formed by the colonic microbiota.

In order to see any effects on weight gain, the rats had to be fed HFD. The weight gain of the rats was on average higher with the HFD than with the LFD, but this effect was partially counteracted by dietary fibre (p = 0.057). When comparing the dietary fibres, pectin had the most noticeable effect, and the effect was greater with a prolonged experimental time. Similar results on rats have been reported elsewhere, with pectin resulting in lower weight gain [4], [40]. Consequently, since the energy content of the HFD was very similar and also the feed intake, the decreasing effect seems to be due to the dietary fibre *per se*. Several hypotheses on why dietary fibre reduces weight gain can be found in the literature, and may be because dietary fibres, especially soluble and viscous ones, have the capacity to slow down and limit food intake or increase satiety and diminish the absorption of nutrients in the small intestine [13], [41]. Another interesting approach is through the SCFAs formed by the microbiota in colon, as suggested by Galisteo *et al* (2008) [7]. A high formation of SCFAs *per se*, is suggested to stimulate PPAR-γ, which increases GLUT-4 and insulin sensitivity. It may be questioned whether all types of dietary fibre giving rise to high amounts of SCFAs have an effect on weight gain, since the differences in weight between groups were quite small. On the other hand, the different fibres gave different formations of SCFAs, which may have an effect. For example, propionic acid has been shown to increase satiety [42], while butyric acid has anti-inflammatory effects through NF-κB, which in turn may influence parameters associated to the metabolic syndrome [42]. It might be questioned whether the high proportion of acetic acid formed by pectin influenced the body weight in this study. In a human study by Johansson *et al* (2013), the consumption of a high-fibre evening meal resulted in less food intake the following day and a greater excretion of breath H_2_ (marker for colonic fermentation), further indicating a role of fermentation in the host metabolism [43].

Dietary fibre increased both the caecal content and the caecal tissue weight in the rats already after 2 weeks on LFD, and many studies have reported similar results when supplementing the diet with dietary fibre [15], [44]. These effects were more pronounced when the experimental time was prolonged to 4 weeks, but no further effects were seen after 6 weeks. The abundance of fat seems to be of less importance, as judged by the similar caecal contents in rats fed corresponding LFD and HFD; an exception was the mixture that gave a considerably higher caecal amount with the HFD than with the LFD. For this specific diet, it may therefore be speculated whether fat also reaches the hindgut of rats. Interestingly, the caecal tissue weight was also higher in rats fed HFD together with the mixture and also with pectin, compared with corresponding LFD. The higher caecal tissue weight might be explained by a higher formation of SCFAs, at least for rats fed the mixture, which had a higher caecal formation of both acetic and propionic acids. These rats also had a high yield of succinic acid, which may also indicate that some fat reached the caecum, thereby influencing the type of SCFA/CA formed. Succinic acid is an intermediary CA, probably formed due to low or changed bacterial activity in colon. An increased caecal concentration of succinic acid (from 0 to 25% of total CAs) and a decreased concentration of butyric acid (from 17 to 4% of total CAs) was seen in rats fed dietary fibre and probiotics 12 days after antibiotic treatment had been stopped [45]. It cannot be excluded that a high abundance of fat in the diet may have similar effects. The average content of butyric acid was also lower in rats fed HFD than those fed LFD, especially with pectin. Not much is known about the function of succinic acid in the colon or the body. Succinic acid has been shown to inhibit motility of the large intestine and to stimulate water secretion from the small intestine [46]. Inagaki *et al* (2007) showed that succinic acid depressed the proliferation rate of the epithelial cells in the colon, as well as reducing the crypt size. Therefore treatment that will give rise to high production of succinic acid should be avoided [46].

The abundance of fat reduced the amount of SCFAs formed in rats fed all the diets, with the exception of the mixture, indicating a lower bacterial activity in the colon. Similar results have been seen in pigs, with high-fat diets suppressing SCFA formation [47]. The reason for this is not known, but it can be speculated whether the microbiota composition is changed due to the high fat content, as shown by Bäckhed (2011) [48], and the formation of SCFAs/CAs may also be changed. One interpretation of this is that high-fat content is harmful in two ways: firstly by being high in energy and therefore leading to overweight and, secondly, by reducing the formation SCFAs, leading to a less healthy colon. Acetic acid has been shown to stimulate proliferation of normal crypt cells and increase colonic motility and blood flow, while propionic acid induces apoptosis of colorectal cancer cells [42]. Patients suffering from ulcerative colitis (UC) were reported to have lower faecal concentrations of butyric acid than healthy subjects [49] and UC patients showed endoscopic and histological improvements after 6 weeks of SCFA rectal irrigations [50]. In a study on patients with UC, β-glucan-enriched oat fibre, known to increase the formation of butyric acid specifically in rats, increased the faecal amounts of butyric acid [51], [52], and symptoms improved simultaneously. Butyric acid has also been shown to decrease mucosal inflammation in UC patients [53]. Even though the formation of SCFAs was reduced with the high-fat content after 2 weeks, the formation increased with a prolonged experimental period, which might indicate an adaption to the diet or that the flora becomes more effective in yielding SCFAs. Other authors have also reported increased SCFA formation with longer experimental period [28]. High concentrations of SCFAs have been shown to decrease gastric emptying rate [54], through the “ileocolonic brake”, which might be important for glycaemic responses and longer satiety. A study on faecal SCFAs in humans fed high-fat diet resulted in higher concentrations of acetic, propionic and butyric acids after 24 weeks [55], which contrasts with the results in the current study. However, it must be kept in mind that the SCFA profiles found in faeces does not necessarily represent the profiles seen in the colon, since approximately 95% of SCFAs are absorbed from the colon [42], [56].

Administration of dietary fibre in LFD for only 2 weeks tended to give lower liver weights (p = 0.078), which is quite surprising. Furthermore, contents of fat, cholesterol and triglycerides in the liver together with dietary fibre were lower than without these components, while there were no differences in plasma cholesterol concentrations between groups. Similar effects could be seen with dietary fibre in HFD, but the effects were much more pronounced. Lower plasma cholesterol concentrations with pectin and guar gum could also be seen in the rats compared with those fed a fibre-free control diet. The differences persisted over time. All this indicates that the consumption of dietary fibre, even at low fat content (5%), has beneficial effect on lipid metabolism. In an attempt to rank the dietary fibres in LFD, the mixture seemed to have most pronounced effects on liver fat, cholesterol and triglycerides (significant for all parameters), followed by guar gum (tendency for cholesterol and p<0.01 for triglycerides) and then pectin (tendency for cholesterol and p<0.05 for triglycerides). Butyric acid and, to some extent, also propionic acid have been suggested to have effects on lipid metabolism [21] and to suppress pro-inflammatory cytokines [7]. Groups fed the mixture and guar gum gave the highest caecal amount of butyric and propionic acids. Furthermore, the caecal amount of butyric acid in relation to that of acetic acid was comparatively higher in rats fed the mixture than the other fibres. However, the formation of the total amount of SCFAs with the mixture was comparatively low compared with other studies using a similar rat model [14]. The reason for that is not known, but it could be due to the comparatively longer time between blood sampling and finishing the animal experiment, as previously suggested by Jakobsdottir *et al* (2013) [57]. A clear colour difference in liver tissue was detected between the groups fed LFD and HFD (data not shown). The colour difference was supported by the higher fat accumulation in the livers of rats fed HFD, and the addition of dietary fibre reduced the fat accumulation for all groups (p<0.05). With HFD it was difficult to rank the effect of fibres, since all dietary fibres had pronounced effects, but pectin seems to have somewhat greater effect than guar gum and the mixture, which both had very similar levels of fat, cholesterol and triglycerides in the liver. The mixture and guar gum gave higher yields of propionic and butyric acids in the rats than pectin. In any case, the results are highly interesting in view of the discussions of obesity and NAFLD, as fat accumulation in the liver is associated with insulin resistance, the metabolic syndrome [58], and type 2 diabetes [59]. A fatty liver has been shown to be insulin resistant and also overproduces glucose, which can lead to hyperglycaemia [60].

After 6 weeks a difference in the spleen weight could be seen when comparing the groups fed guar gum and the fibre-free diets, where the fibre-free gave larger spleens (p = 0.043). A higher spleen weight has been proposed to be a marker for systemic inflammation [61], [62]. However, no differences were seen in rats fed LFD and HFD at 2 and 4 weeks, and it seems that triggering inflammation in conventional rats with fat probably takes some time and requires longer exposure to HFD, such as 6 weeks. However, the level of inflammation, measured as MCP-1 (p<0.05), was lower in groups fed dietary fibre in LFD already after 2 weeks, especially with guar gum, and might be a more sensitive marker for inflammation than the weight of the spleens. This type of fibre also had an effect in HFD and also at a prolonged experimental time (correlation factor 0.56, p = 0.013). Obesity is now often defined as an inflammatory condition, resulting in a chronic inflammation which later in life can cause other disorders like insulin resistance or type 2 diabetes [63]. These results are interesting as dietary interventions engaging obese subjects in the risk zone of developing insulin resistance or type 2 diabetes could have a great effect on their metabolic health.

Interestingly, the microbiota composition in groups fed pectin, guar gum and the fibre-free diet was completely different, indicating that the different types of dietary fibre affect the composition of the microbiota. When the gut microbiota, represented by the TRFs, of the individual rats were linked to specific analytical parameters, guar gum gave abundance of *Bacteroides*, which was also linked to butyric acid both in serum and caecum, as well as to the caecal content of propionic acid. *Bacteroides* has been shown to form both acetic and propionic acids in the colon [42]. The rats fed guar gum had also a high abundance of other bacteria, which were also connected to the SCFAs. Interestingly, in the group fed the fibre-free diet, the microbiota composition was more linked to negative parameters like liver cholesterol, triglyceride and inflammation and *Akkermansia*. This is in agreement with another study, which also showed that *Akkermansia* grows well in the colon of rats fed high-fat diets (unpublished results, Zhong Y, Fåk F, Nyman M). It has also been suggested that *Akkermansia* could play a role in the disease development of inflammatory bowel disease and that mucin degradation by *Akkermansia* facilities intestinal inflammation in *Salmonella* Typhimurium infected mice [64]. Furthermore, a metagenomic study on subjects with type 2 diabetes showed enrichment of some genes belonging to *Akkermansia* [65]. The opposite was seen in mice fed high-fat diets and prebiotics (oligofructose) in a study by Everard *et al* (2013), where the number of *Akkermansia* was negatively correlated with body weight and the abundance decreased in obese and diabetic C57BL/6J mice [66]. These conflicting data have to be clarified. It should be noted that the control group in this study were fed a high fat diet without any dietary fibre. *Akkermansia* may degrade mucin, and under the extreme situation existing in the colon without any available polysaccharides, it is perhaps not surprising that the number of *Akkermansia* increased. Further, in contrast to the study by Everard *et al* [66], who used C57BL/6 mice disposed to develop insulin resistance and atheroscleorosis when fed a high-fat diet, conventional rats (i.e. healthy rats) were used in this study. This may be a plausible explanation to the different results, since high-fat diets without any fibre were used in both the present study and in the study be Everard *et al* [66]. No bacterial taxa linked to the groups fed pectin could be identified, but this group gave particularly high concentrations of acetic acid. Greater individual variation was seen in the rats fed pectin than those fed guar gum or the fibre-free diet, indicating that pectin had a less selective effect on the microbiota than guar gum or fat alone without fibres.

In conclusion, supplementation with fermentable dietary fibre affected weight gain, fat content of the liver and also the amount of cholesterol and triglycerides in the liver. The effects were more pronounced in HFD than in LFD and the differences increased with time. Pectin and the mixture seem to be especially prone to lower these parameters. Dietary fibre also seemed to decrease systemic inflammation, as judged by the lower MCP-1 values and lower spleen weights. High-fat content in the diet resulted initially in a reduced formation of SCFAs in caecum and in the circulation, but gradually recovered after a longer experimental period. There was an increase in concentration of succinic acid in rats fed a HFD and it increased markedly with a prolonged experimental time, while butyric acid concentration decreased, indicating a change in the caecal microbiota composition. However, guar gum and the mixture counteracted this effect and stimulated the butyric acid formation after 6 weeks. A correlation between the caecal microbiota and SCFA, liver cholesterol and triglycerides, inflammatory cytokine and pH was seen. It might therefore be speculated whether some microbial metabolites could serve as markers for e.g. cholesterol metabolism or SCFA formation.
